# Ready‐To‐Use and Rapidly Biodegradable Magnesium Phosphate Bone Cement: In Vivo Evaluation in Sheep

**DOI:** 10.1002/adhm.202300914

**Published:** 2023-06-04

**Authors:** Lena Schröter, Friederike Kaiser, Anna‐Lena Preißler, Philipp Wohlfahrt, Oliver Küppers, Uwe Gbureck, Anita Ignatius

**Affiliations:** ^1^ Institute for Orthopedic Research and Biomechanics Ulm University Medical Center Helmholtzstraße 14 D‐89081 Ulm Germany; ^2^ Department for Functional Materials in Medicine and Dentistry University Hospital Würzburg Pleicherwall 2 D‐97070 Würzburg Germany

**Keywords:** bone cements, large animal models, magnesium phosphates, ready‐to‐use, resorbable

## Abstract

In clinical practice, hydroxyapatite (HA) cements for bone defect treatment are frequently prepared by mixing a powder component and a liquid component shortly before implantation in the operation theater, which is time‐consuming and error‐prone. In addition, HA cements are only slightly resorbed, that is, cement residues can still be found in the bone years after implantation. Here, these challenges are addressed by a prefabricated magnesium phosphate cement paste based on glycerol, which is ready‐to‐use and can be directly applied during surgery. By using a trimodal particle size distribution (PSD), the paste is readily injectable and exhibits a compressive strength of 9–14 MPa after setting. Struvite (MgNH_4_PO_4_·6H_2_O), dittmarite (MgNH_4_PO_4_·H_2_O), farringtonite (Mg_3_(PO_4_)_2_), and newberyite (MgHPO_4_·3H_2_O) are the mineral phases present in the set cement. The paste developed here features a promising degradation of 37% after four months in an ovine implantation model, with 25% of the implant area being newly formed bone. It is concluded that the novel prefabricated paste improves application during surgery, has a suitable degradation rate, and supports bone regeneration.

## Graphical abstract

An injectable, ready‐to‐use magnesium phosphate bone cement based on glycerol is developed. After application, the glycerol in the cement is exchanged against water from the surrounding tissue. Ex vivo in pig tibia bone, the cement hardens rapidly within 24 h. Excellent biocompatibility and a cement degradation of 37% after four months are observed in vivo in sheep.

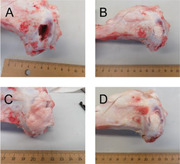

## 1 Introduction

In general, bone has considerable potential for regeneration, being one of the few tissues that can heal without scar formation.^[^ ^1^ ^]^ However, large bone defects resulting, for example, from trauma, tumors, or infection, require surgical therapy, because spontaneous regeneration is limited to relatively small defects.^[^ ^2^ ^]^ Currently, autologous bone transplantation into the defect is still considered to be the gold standard, despite important limitations, including availability, donor site morbidity, and long operation times.^[^ ^3^ ^]^

Researchers have been working for decades to develop synthetic bone substitutes to overcome the use of autologous material. Calcium phosphates, such as cold‐setting hydroxyapatite (HA) cements, chemically resemble the inorganic component of natural bone and are already established in the clinic as synthetic bone substitute materials.^[^ ^4^, ^5^ ^]^ However, HA cements have the disadvantage of slow degradation, and in some cases cement residues are still present at the implant site years after implantation, increasing the risk for late bone fractures around the implant.^[^ ^6^, ^7^, ^8^, ^9^ ^]^ To achieve a restitutio ad integrum as in natural bone healing of small defects, a bone cement is required that degrades completely within a few months, ideally synchronously with new bone formation.^[^ ^10^ ^]^

Bone substitutes based on mineral magnesium phosphate represent a promising advancement on calcium phosphate cements, because they degrade more rapidly in vivo and have a similarly good biocompatibility.^[^ ^11^, ^12^, ^13^ ^]^ The best known and most studied magnesium phosphate phase to date is struvite (MgNH_4_PO_4_·6H_2_O).^[^ ^12^ ^]^ Cold‐setting struvite cements are normally prepared by mixing trimagnesium phosphate (Mg_3_(PO_4_)_2_, TMP) or magnesium oxide with diammonium hydrogen phosphate ((NH_4_)_2_HPO_4_, DAHP) solution or a solution containing DAHP and ammonium dihydrogen phosphate (NH_4_H_2_PO_4_).^[^ ^13^ ^]^ Instead of pure TMP, the use of a calcium magnesium phosphate (e.g., Ca_0.25_Mg_2.75_ (PO_4_)_2_) as a raw powder is also possible.^[^ ^14^ ^]^

Struvite exhibits a higher passive solubility compared to HA under physiological conditions, the latter being limited to the in vivo resorption by macrophages or osteoclasts.^[^ ^15^ ^]^ Two in vivo studies, including one of our own, demonstrated that struvite cements degrade overall more rapidly compared to (calcium deficient) HA and brushite cements.^[^ ^16^, ^17^ ^]^ Although the ammonium ions are sometimes critically discussed in terms of possible toxicity, the material, to date, has proved to be biocompatible as well as osteoconductive, and the resorbed cement was replaced by bone tissue in several studies.^[^ ^16^, ^18^, ^19^, ^20^ ^]^ Brushite (CaHPO_4_·2H_2_O) and monetite (CaHPO_4_) are calcium phosphates that, similarly to struvite, have also a higher passive, non‐cell‐mediated solubility compared to HA cements.^[^ ^21^ ^]^ However, the transformation of brushite into low soluble calcium phosphates like HA or octacalcium phosphate was observed in several in vivo studies, reducing the degradation rate in long‐term.^[^ ^17^, ^22^, ^23^, ^24^ ^]^

Most of the current clinically approved bone cements are manually mixed in the operating theater from powder and aqueous cement liquid. This immediately initiates the cement setting reaction and restricts the surgeon to applying the cement in a time window of only a few minutes after mixing. Greater time saving and flexibility for clinical use would be achieved with a premixed cement paste that is storage‐stable and ready‐to‐use without the need for mixing during surgery.^[^ ^25^ ^]^ These pastes normally consist of the powder components of the cement and a liquid that does not lead to a setting reaction and is later exchanged by water from the surrounding body tissue after implantation.^[^ ^25^ ^]^ This enables the water‐based cement reaction to occur after application, resulting in cement setting. Such premixed paste systems have been studied, to date, to a limited extent for calcium phosphates, with raw powders being either 1a) tetracalcium phosphate and dicalcium phosphate, 1b) *α*‐tricalcium phosphate (*α*‐TCP), or 1c) *α*‐TCP, dicalcium phosphate, and calcium carbonate for the reaction to HA or 2) *β*‐TCP and monocalcium phosphate for the reaction to brushite.^[^ ^26^, ^27^, ^28^, ^29^, ^30^, ^31^, ^32^, ^33^, ^34^, ^35^, ^36^, ^37^ ^]^ Glycerol, polyethylene glycol, and Miglyol 812 (triglyceride) in combination with two surfactants, or pyrophosphate decahydrate have, to date, been investigated as unreactive cement liquids.^[^ ^26^, ^27^, ^28^, ^29^, ^30^, ^31^, ^32^, ^33^, ^34^, ^35^, ^36^, ^37^ ^]^ A HA‐forming premixed paste with Miglyol and surfactants is already clinically available.^[^ ^31^ ^]^

In contrast to prefabricated calcium phosphate cements, which are studied to a limited extent, prefabricated magnesium phosphate cements are virtually unstudied. To the best of our knowledge, only one publication has investigated a struvite‐forming prefabricated magnesium phosphate paste, in which calcium magnesium phosphate powder, ammonium dihydrogen phosphate powder, Miglyol, and surfactants were combined to form a paste.^[^ ^14^ ^]^ The paste was not applied directly into the investigated drillhole defects in rabbits, but mixed beforehand with 0.5 m dipotassium hydrogen phosphate solution using a double‐chamber syringe. Both cement pastes, based on Ca_0.25_Mg_2.75_(PO_4_)_2_ or Ca_0.75_Mg_2.25_(PO_4_)_2_, revealed complete osseointegration, good biocompatibility, and no formation of soft connective tissue. Decreased implant size, a fissured surface of the samples, and decreased X‐ray opacity indicated a more rapid resorption of the two struvite‐forming pastes compared to the HA‐forming reference paste, but the degradation was not quantified.^[^ ^14^ ^]^

In the present study, we combined the promising rapid resorption of struvite‐forming, cold‐setting bone cements with the application advantages of premixed pastes, including a broader application window during operation, avoided personal influence of the person mixing the cement during the operation, and reduced contamination risk. A premixed magnesium phosphate paste consisting of TMP (Mg_3_(PO_4_)_2_), DAHP (NH_4_)_2_HPO_4_), and glycerol (C_3_H_8_O_3_) was developed. Due to the hygroscopic properties of glycerol, no additional surface modifiers need to be added to the prefabricated paste to enable the exchange with water. Pure TMP was chosen because it exhibits an increased reactivity compared to calcium magnesium phosphate, which results in a more rapid cement hardening. Additionally, the cement was applied directly, as a one‐component system into the defect, without the need for prior mixing with an aqueous solution as in the study of Ewald et al.^[^ ^14^ ^]^ This extends the surgeon's application window from a few minutes to an unlimited time.

We hypothesized that injectability and the injection force of the premixed magnesium phosphate paste can be optimized by using a multi‐modal particle size distribution (PSD) within such pastes. Both application‐ and degradation‐relevant properties, such as storage stability of the cement paste, and the compressive strength and porosity of the hardened cement were investigated. We further hypothesized that the premixed cement paste exhibits a similar high biocompatibility and degradation profile in vivo as the “classical” two‐component struvite cement. This hypothesis was investigated in a sheep model, and the degradation was quantified via histomorphometry.

## 2 Experimental Section

### 2.1 Raw Powder Synthesis

TMP (farringtonite, Mg_3_(PO_4_)_2_) was synthesized by mixing MgHPO_4_·3H_2_O (Alfa Aesar, Ward Hill, USA) and Mg(OH)_2_ (VWR International, Radnor, USA) in a molar ratio of 2:1 and sintering for 5 h at 1100 °C. The resulting sinter cake was crushed and sieved to reach particle sizes of ≤355 µm. To obtain coarse TMP, 125 g of the powder was milled with four zirconia grinding balls (*Ø* = 25 mm) for 1 h with a planetary ball mill PM 400 (Retsch, Haan, Germany). For fine TMP, 125 g of the powder was milled with 100 zirconia grinding balls (*Ø* = 7–10 mm) for 16 h in 125 mL ethanol with the same planetary ball mill. The wet‐milled powder was subsequently dried in a fume hood.

### 2.2 Cement Fabrication

Pastes were fabricated by mixing 1.5 g DAHP (Sigma Aldrich, St. Louis, USA) with 6 g TMP and 3.2 g glycerol (Carl Roth GmbH & Co. KG, Karlsruhe, Germany), resulting in a PLR of 2.95 g mL^−1^. The amount of total TMP was kept constant, whereas the ratio between the fine and coarse TMP was varied (**Table** **1**). The percentage in the paste labels (0%, 20%, and 80%) refers to the content of the fine TMP related to the total powder content in the paste. The DAHP was ground for 20 s in a coffee grinder before adding to the paste. The mixing of the paste was conducted twice in a planetary centrifugal mixer ARV‐310 CE (Thinky, Laguna Hills, USA) for 20 s at 200 rpm and for 3 min at 2000 rpm at atmospheric pressure. In the in vivo study, the 20%‐paste was investigated.

**Table 1:** Composition of the magnesium phosphate cement pastes. The (weight) percentage (0%, 20%, and 80%) refers to the content of the fine TMP related to the total powder content in the paste

Paste	Coarse TMP [g]	Fine TMP [g]	DAHP [g]	Glycerol [g]
0%‐paste	6	0	1.5	3.2
20%‐paste	4.5	1.5	1.5	3.2
80%‐paste	0	6	1.5	3.2

### 2.3 Particle Size

The particle size of the raw powders after milling in the planetary ball mill/coffee grinder was determined by laser diffraction analysis (Horiba LA 300 Wet, Horiba, Kyōto, Japan) in triplicate. Before the measurement, the powder was suspended in isopropanol in an ultrasonic bath.

### 2.4 Injection Force and Injectability

Directly after mixing of the paste or after 4 weeks of paste storage, injection force and injectability were both determined in triplicate. For this purpose, three 5 mL Luer lock syringes were weighed empty for each paste (*m*_1_), each filled with ≈5 g paste (*m*_2_) and weighed again after the injection test (*m*_3_). Injectability (*I*) was calculated according to Equation (1).

(1) $$I=\left(1-\frac{m_{3}-m_{1}}{m_{2}}\right)\times 100\%$$

The syringe outlet diameter of the 5 mL Luer lock syringe was 2 mm, and no canula was used. The injection was performed with a universal testing machine (Z010, Zwick Roell, Ulm, Germany) with a 10 kN load cell. The compression die was pressed with a velocity of 300 mm min^−1^ on the syringe plunger. The upper force limit was set to 350 N. The injection force was determined as the maximum force measured in the plateau region of the force–displacement curve during injection. If the pastes were stored before injection force and injectability measurement, this was performed in 100 mL polypropylene sample containers with lid or glass containers with crimp caps. Pastes were stirred by hand after storage before transferring to the syringe.

### 2.5 Cohesion

For the cohesion test, the cement paste was injected into 50 mL ultrapure water in a petri dish. Images were obtained directly after injection or after 24 h of storage at 37 °C, during which the petri dish was covered with a lid.

### 2.6 Compressive Strength

Compression tests were conducted at the same universal testing machine and with the same load cell as used for the injection force measurement. Rectangular‐shaped samples (6 × 6 × 12 mm^3^, *n* ≥ 8) were fabricated by filling of the cement paste into silicone molds and storage of the molds for 1, 3 or 7 days in ultrapure water at 37 °C. After setting, the hardened cement samples were removed from the molds and finished with sandpaper. Samples were measured upright with a compression velocity of 1 mm min^−1^.

### 2.7 Phase Composition

The phase composition was investigated by powder XRD with a D8 Advance (Bruker AXS, Karlsruhe, Germany). The in vitro samples were crushed with pestle and mortar after 3 or 7 days of setting in ultrapure water at 37 °C. The resulting powder was placed in poly(methyl methacrylate) (PMMA) sample holders. In the case of paste measurements, the paste was directly placed in the PMMA sample holder. The measurements were performed using copper K_*α*_‐radiation, a power of the X‐ray tube of 1600 W, and a divergence slit of 2.5°. Samples were measured in an angle range of 7–70° (2Theta) and with a step size of 0.02°, a dwell time of 0.6 s/step, and a rotation speed of 15 rpm. The quantitative phase composition was determined with Rietveld refinement using the software TOPAS 6 (Bruker AXS). For powder refinement, the structures ICSD #5148 (dittmarite), COD #9007674 (struvite), COD #9012534 (farringtonite), and COD #9007632 (newberyite) were used. For the in vivo samples, fragments of implanted material (*n* = 3) were dried at 37 °C and crushed with pestle and mortar. Non‐implanted reference material (*n* = 3) of the 20%‐paste was prepared as described above. XRD measurements were also performed as described above, except that the powder samples were placed in silicon sample holders and the dwell time was increased to 1 s/step. For phase analysis, additionally to the abovementioned ones, the structures ICSD #202099 (baricite), ICSD #202098 (bobierrite), and ICSD #51490 (Mg_3_(PO_4_)_2_·4H_2_O) were used.

### 2.8 pH Value

The pH value (inoLab pH meter, Xylem Inc., Washington, US) was measured for 12 h, with a measurement point every 30 min and automatic recording. Before the start of the measurement, 2 mL prefabricated paste were injected into 10 mL phosphate buffered saline and the pH electrode was placed within the hardening cement paste.

### 2.9 Porosity

Mercury porosimetry (Pascal 140/440, Porotec, Hofheim, Germany) was applied to determine the porosity and mean pore diameter of cement samples made of the 20%‐paste composition which were hardened for 3 days at 37 °C in ultrapure water and dried for 12 h at 37 °C. The Washburn Equation (2) allowed for the calculation of the pore radius *r* from the applied pressure *p*, assuming a mercury contact angle *θ* of 140° and a surface tension *γ* of 0.48 N m^−1^. The relative pore volume could be calculated because at each pressure step the mercury volume was determined. The measurement was performed in triplicate.

(2) $$r=\frac{2\gamma \cos\theta }{p}$$

### 2.10 FTIR Measurements

FTIR measurements were performed of pure glycerol, of the fresh 20%‐paste, and after 3 or 7 days of setting in ultrapure water at 37 °C. The cement samples were directly crushed with pestle and mortar after removing from the incubator and the resulting powder was measured with a Nicolet iS10 with smart iTR diamond ATR (attenuated total reflectance, Thermo Fisher Scientific, Waltham, USA).

### 2.11 Ex Vivo Experiment

Porcine tibia bones were obtained from a local abattoir to analyze whether the cement paste hardens ex vivo in bone tissue. A wedge defect with a similar size compared to the in vivo study (height: 6 mm, width: 14 mm, length: 24 mm) was created in the tibia plateau and the cement paste was filled into the defect and covered with soft tissue. The bone was wrapped in wet paper towels and incubated for 24 h at 37 °C and 100% humidity. Subsequently, the paper towels and the soft tissue were removed, and it was determined whether the cement had hardened.

### 2.12 In Vivo Study

The animal experiment was approved by the local ethical committee (Regierungspräsidium Tübingen, Germany, no. 1451). All animal procedures were performed in accordance with the European Union Directive 2010/63/EU on the protection of animals used for scientific purposes. In total, 14 adult female merino sheep (age: 4–6 years, mean weight: 97 ± 9 kg), including two reserve animals, underwent surgery and received the prefabricated cement (20%‐paste) randomly in one hind limb. For implantation, a well‐established mechanically loaded, clinically relevant defect model at the proximal tibia was used, which is described in detail elsewhere.^[^ ^38^, ^39^ ^]^ The model was suggested to mimic clinical situations in trabecular regions with moderate load bearing, for example, at tibia plateau or distal radius fractures.^[^ ^40^ ^]^ To reduce the numbers of animals according to the 3Rs principle, a control group with empty defects was omitted in the current study, because the defect model was previously shown to be of critical size.^[^ ^18^ ^]^ The implantation of a clinically established reference cement was also omitted deliberately, because a CDHA cement as a clinical standard had already been investigated in a previous study in the same defect model using the same experimental procedure.^[^ ^18^ ^]^ One animal was excluded from the study because of an anesthetic incident during surgery.

### 2.13 Surgical Procedure

All surgical interventions were conducted under general anesthesia, induced by thiopental (5 mg kg^−1^ body weight; Thiopental Inresa, Inresa GmbH, Freiburg, Germany) intravenously (i.v.) and maintained by inhalation of isoflurane (Isofluran Baxter, Baxter GmbH, Unterschleißheim, Germany) in oxygen. A medial approach was used to gain access to the knee region and the proximal tibia. Here, a standardized wedge‐shaped bone defect (height: 6 mm, width: 14 mm, length: 24 mm) was milled into the tibial bone 3 mm beneath and parallel to the medial tibial plateau under saline irrigation, using a custom‐made machine.^[^ ^41^ ^]^ Following the removal of bone debris, the prefabricated cement paste was applied into the bone defect. Thereafter, the wound was routinely closed in layers. Perioperatively and for 3 days postoperatively each animal received carprofen (4 mg kg^−1^ body weight s.c.; Rimadyl, Zoetis GmbH, Germany) and amoxicillin trihydrate (10 mg kg^−1^ body weight s.c.; Veyxyl LA 20%, Veyx‐Pharma GmbH, Schwarzenborn, Germany) to provide analgesia and antibiosis, respectively.

### 2.14 Fluorochrome Labeling

To assess new bone formation dynamically, fluorochrome labeling was performed with a time interval of 14 days. At 4 (2 months group, *n* = 6) or 11 weeks (4 months group, *n* = 7) post‐surgery, each animal received a fluorochrome bone label injection of tetracycline hydrochloride (25 mg kg^−1^ body weight, i.v.; Ursocyclin 10% pro inj., Medistar Arzneimittelvertrieb GmbH, Ascheberg, Germany). This was followed by a second bone label injection of calcein green (10 mg kg^−1^ body weight, i.v.; Sigma‐Aldrich, Merck KGaA, Darmstadt Germany) after 6 weeks (2 months group, *n* = 6) or 13 weeks (4 months group, *n* = 7). After 2 and 4 months, the animals were killed and the tibiae with the implanted struvite cement pastes were harvested for histological analysis.

### 2.15 Histology

Briefly, the defect regions with the implanted prefabricated cement pastes were dissected with ≈10 mm of surrounding bone and divided into two parts. While the medial part was used for non‐decalcified histology, the lateral part was processed for decalcified histology. After 5 days of fixation in 4% buffered formaldehyde, the medial part of the tibial specimens was dehydrated in an ascending series of ethanol for non‐decalcified histology, as described in more detail elsewhere.^[^ ^40^ ^]^ Thereafter, the samples were embedded in methyl methacrylate (Merck KGaA) and ground sections of ≈90–110 µm were prepared, using the cutting–grinding technique of Donath and Breuner.^[^ ^42^ ^]^

### 2.16 Fluorescence Microscopy and Bone Formation Rate Determination

Subsequently, the sections were evaluated with a light microscope (Leica DMI6000B, Heerbrugg, Switzerland) using fluorescent light (filter cubes LED 405 and L5 ET for an excitation wavelength of 402 and 494 nm, respectively, both from Leica). To determine the BFR in the region where newly formed bone was replacing the degrading cement paste, the spatial distribution of the yellow tetracycline‐ and green calcein‐labeled bone was assessed in a rectangular region of interest (ROI) of ≈11 mm^2^ adjacent to the proximal cement surface (*n* = 6 for the 2 months group, *n* = 7 for the 4 months group). Analysis was performed using histomorphometrical software (Osteomeasure, Osteometrics Inc., Decatur, GA, USA) according to the guidelines of the American Society for Bone and Mineral Research.^[^ ^43^ ^]^

### 2.17 Histological and Histomorphometrical Evaluation

Following fluorescent microscopy, the surfaces of the ground sections were stained with Giemsa for qualitative and quantitative histological evaluation according to standard protocols. The samples were evaluated in a blinded fashion. For quantitative histomorphometrical assessment (*n* = 6 for the 2 months group, *n* = 7 for the 4 months group), the stained sections were scanned with a light microscope (Leica DMI6000B) at 50‐fold magnification. The relative amount of the residual cement paste (Cm.Ar/T.Ar), newly formed bone (B.Ar/T.Ar), and soft tissue (ST.Ar/T.Ar) was determined in a 7.0 mm × 7.0 mm ROI in the center of the defect area, using histomorphometrical software (Leica MMAF 1.4.0 MetaMorph Imaging System, Leica, Wetzlar, Germany), as described previously.^[^ ^44^ ^]^

### 2.18 TRAP Staining

For decalcified histology, the tibial specimens were decalcified in ethylenediaminetetraacetic acid for 3 months, embedded in paraffin, cut into 7 µm sections, and stained for TRAP to investigate cellular resorption of the implanted cement paste. TRAP‐positive cells with ≥3 nuclei, residing on the bone or cement paste surface, were defined as osteoclasts. Osteoclastic cells were counted in six visual fields at the bone‐cement interface under 200‐fold magnification to determine the number of osteoclasts on the cement surface (N.Oc/Cm.Pm) using Osteomeasure software.

### 2.19 Statistics

Numerical data were presented as mean ± standard deviation, with the error bars in figures representing the standard deviation. For statistical analysis of the compressive strength data, one‐way analysis of variance (Holm–Sidak) was performed to compare groups within the same paste storage time (0 days) and within the same cement setting time (3 days) with the software SigmaPlot (Systat Software GmbH, Düsseldorf, Germany). The data passed the normality test (Shapiro–Wilk) and the equal variance test, performed with the same software. Preceding the animal experiment, the required sample size was calculated by a statistician at the Institute of Epidemiology and Medical Biometry, Ulm University. Statistical analyses of data obtained in the animal experiment were performed using GraphPad Prism (8.4.3, GraphPad Software Inc., La Jolla, CA, USA). All in vivo data were tested for normal distribution by Shapiro–Wilk test. Normally distributed data (B.Ar/T.Ar, N.Oc/Cm.Pm) was analyzed using unpaired *t*‐test. Where normal distribution was not given (Cm.Ar/T.Ar, ST.Ar/T.Ar, BFR), data was tested by Mann–Whitney test. The level of significance was set at *p* < 0.05.

## 3 Results and Discussion

### 3.1 Physicochemical Characterization

#### 3.1.1 Particle Size Distribution, Injection Force, and Injectability

**Figure 1:**
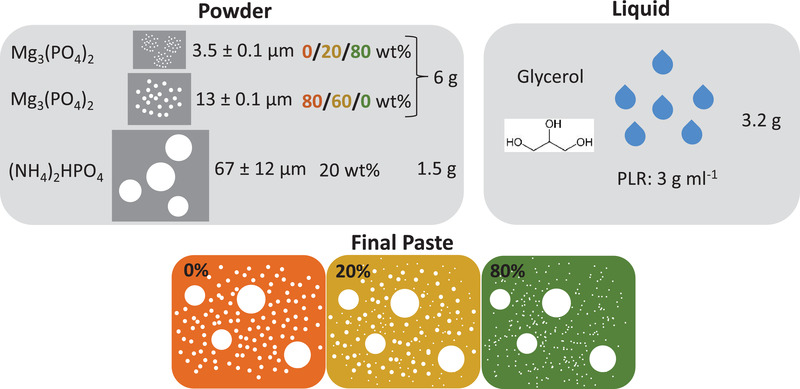
Composition of three different ready‐to‐use magnesium phosphate bone cement pastes. The cement paste hardens by the in vivo exchange of glycerol against H_2_O coming from the surrounding tissue. The DAHP content and the PLR were kept constant between the three pastes, only the ratio between fine and coarse grained TMP was varied. The percentage in the final paste labels refers to the content of the fine TMP related to the total powder content in the paste.

**Figure 2:**
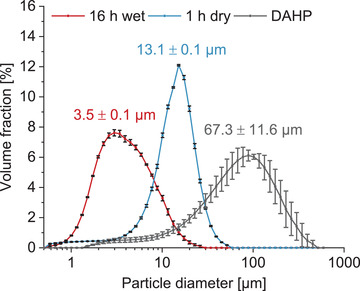
PSD and median particle diameter of the three raw powders used for the paste fabrication: fine Mg_3_(PO_4_)_2_ (16 h wet milled), coarse Mg_3_(PO_4_)_2_ (1 h dry milled), and (NH_4_)_2_HPO_4_ (DAHP), *n* = 3. All three raw powders exhibited monomodal PSDs resulting in a trimodal PSD in the cement paste with 20% fine Mg_3_(PO_4_)_2_.

In the current study, we investigated three different paste compositions regarding their influence on the injection force and injectability of a prefabricated magnesium phosphate cement paste: Two bimodal PSDs, including DAHP and either a coarsely ground TMP (13 µm) or a finely ground TMP (3.5 µm), and one trimodal PSD combining all of them (**Figures** **1** and **2**). The different paste compositions were labeled according to the content of fine TMP in the powder: The 0%‐paste contained 80% coarse TMP and 20% DAHP and the 80%‐paste contained 80% fine TMP and 20% DAHP. The 20%‐paste included 20% fine TMP, 60% coarse TMP, and 20% DAHP, and therefore exhibited a trimodal PSD (Figure 1). In all the compositions, the powder‐liquid‐ratio (PLR) of 2.95 g mL^−1^ and the DAHP content of 20 wt%, referring to the total powder weight, were kept constant. The DAHP content corresponded to a “classical” non‐prefabricated struvite cement prepared with 3.5 m DAHP solution and a PLR of 2 g mL^−1^. Only the particle size (distribution) of the TMP raw powder was changed in the premixed paste.

**Figure 3:**
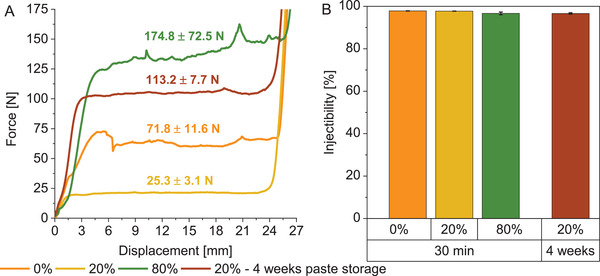
A) Injection force and B) injectability of different paste compositions with preceding paste storage of 30 min or 4 weeks, *n* = 3. Only one representative curve of the three measurements is displayed for the injection force. The (weight) percentage refers to the content of the fine TMP related to the total powder content in the paste. The paste with a trimodal PSD (20%‐paste) exhibited the lowest injection force.

Injectability was similar for all three pastes, with values between 96% and 97% (**Figure** **3**B). All pastes were fully injected, the missing 3–4% being the paste residues in the syringe head that could not be pushed out by the plunger. Filter pressing was not observed in any paste. The injection force was noticeably higher for both bimodal pastes of 175 N (80% fine TMP) and 72 N (0% fine TMP) compared to 25 N for the paste with a trimodal PSD (Figure 3A).

Commercially available bone cements are normally either injected with a syringe or, in the case of a putty‐like consistency, directly molded into the defect by hand. For application by syringe, a high injectability and a maximum injection force of 200 N are required.^[^ ^45^, ^46^ ^]^ Ideally, the injectability is as close as possible to 100%, which means that the entire cement paste is extruded. The “filter pressing” phenomenon, that is, the separation of powder and liquid within the syringe, must be avoided.^[^ ^47^ ^]^ At the same time, the paste should be sufficiently viscous that it does not flow directly out of the syringe. Injectability of cement pastes can be improved by a bimodal PSD of the raw powder, which means that there are two maxima in the PSD.^[^ ^45^ ^]^ This increases the packing density of the powder, thereby reducing the volume of liquid necessary to fill the pores between the particles.^[^ ^45^ ^]^ The resulting excess liquid at a constant PLR increases the distance between the particles and improves flowability.^[^ ^45^ ^]^

According to O'Neill et al., generally a bimodal PSD is advantageous for injectability and injection force of cement pastes compared to a monomodal PSD.^[^ ^45^ ^]^ The authors stated that the ideal bimodal PSD to achieve the highest packing density consists of 25 vol% fine particles and 75 vol% large particles.^[^ ^45^ ^]^ However, a higher packing density of a bimodal PSD compared to a monomodal PSD is only achievable if the size difference between the particles is large enough.^[^ ^45^ ^]^ The authors did not specify the necessary size difference, but the fine particles have to fit in the voids between the large particles.^[^ ^45^ ^]^ In the present study, for the 0%‐paste and the 80%‐paste, the content of small particles (fine or coarse TMP) was 70 vol% and the content of large particles (DAHP) was only 30 vol%. This TMP/DAHP ratio was chosen because it is equivalent to a similar cold‐setting, non‐prefabricated struvite cement fabricated with a 3.5 m DAHP solution and a PLR of 2 g mL^−1^. For the packing density, however, this ratio is not ideal and results in the so‐called “loosening effect,”^[^ ^45^ ^]^ which means that the high TMP content disturbs the powder network of the DAHP. For the 80%‐paste, there is likely the additional effect of agglomeration of the fine TMP, which further increases the injection force.

The trimodal PSD (20%‐paste), by contrast, led to a considerable reduction of the injection force to 25 N (Figure 3A). Because the DAHP/TMP ratio was the same, this could only have been caused by the bimodal PSD of the TMP. The fine TMP content of 20 wt%, based on the total powder mass including DAHP, resulted in a proportion of 25 vol% if only the TMP was considered. This corresponded, at least for the TMP, in the abovementioned ideal content of fine particles for a high packing density in a bimodal PSD. Because the PLR was kept constant, the higher packing density compared to the 0%‐ and 80%‐paste likely resulted in a better flowability and, therefore, a lower injection force. The effect might be further improved by decreasing the particle size of the fine TMP, because the size difference between the fine and coarse TMP, which was a factor of 3.7 in diameter, might be too small to increase the packing density optimally.

**Figure 4:**
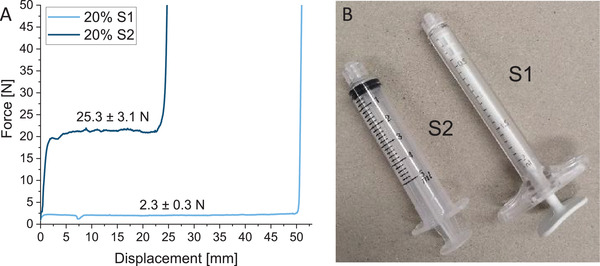
A) Representative curves of the injection force of the 20%‐paste composition measured with B) two different syringe types. The injection force (*n* = 3, maximum force in the plateau region) decreased approximately tenfold by using a syringe with broader syringe “wings”. Diameter S1: 10.6 mm, S2: 13.6 mm, outlet diameter S1: 3 mm, S2: 2 mm. For the injection force measurements in Figure 3, the S2‐syringe was used.

It is important to note that the injection force strongly depends on the syringe geometry, particularly the syringe diameter, the outlet diameter, and the used needle. The maximum force that can be manually applied also depends on the ergonomic design of the syringe. By using a syringe with a smaller diameter and a larger outlet diameter, the injection force of the 20%‐paste‐composition was decreased approximately tenfold (**Figure** **4**). Additionally, the broader syringe “wings” of the S1‐syringe strongly improved the hand ergonomics during injection.

#### 3.1.2 Paste Cohesion

**Figure 5:**
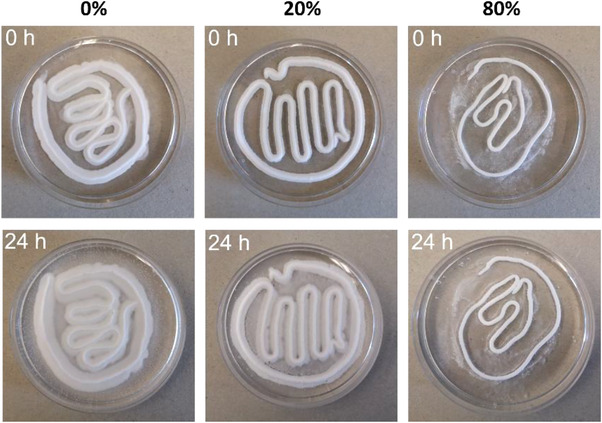
Cohesion of different paste compositions directly after injection (0 h) into ultrapure water and 24 h after injection and storage at 37 °C, *n* = 3. The (weight) percentage refers to the content of the fine TMP related to the total powder content in the paste. Cohesion improved with increasing content of fine TMP powder. Petri dish diameter was 8.3 cm.

High cohesion of the cement paste in liquids is desirable, because the cement properties are less affected in case of blood contact. For the cement paste cohesion in water, the bimodal PSD with the fine TMP particles (80%‐paste) was found to be the optimum (**Figure** **5**). In general, it was found that the larger the proportion of fine TMP particles, the better the cohesion. It was observed during paste preparation that the 80%‐paste had a higher viscosity than the other two pastes, likely contributing to a better cohesion in water. It is already known from calcium phosphate pastes that the viscosity behaves inversely to the particle size.^[^ ^45^, ^48^ ^]^

With respect to the 0%‐paste, water can also presumably penetrate more readily into the larger pores between the coarse TMP powder and wash particles out of the paste. In addition, it can be assumed that due to the higher reactivity of the fine TMP, the edge of the paste reacts more rapidly when it comes into contact with water. The paste hardened at the edge possibly forms a barrier that prevents strong diffusion of water into the paste.

#### 3.1.3 Phase Composition, pH Value, Porosity, Compressive Strength, In Vitro Setting in Bone, and Storage Stability

**Figure 6:**
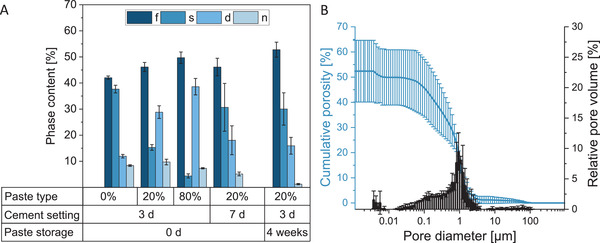
A) Quantitative phase composition determined by XRD measurement and Rietveld refinement after setting of the cement pastes in ultra‐pure water for 3 or 7 days at 37 °C, *n* = 3. The “4 weeks”‐paste was stored for 4 weeks before the 3‐day‐hardening step in ultrapure water at 37 °C. f‐farringtonite, s‐struvite, d‐dittmarite, n‐newberyite. The premixed pastes set to dittmarite, struvite, and newberyite. B) Pore size distribution and porosity of the paste with 20% fine TMP content after 3 days of setting in ultrapure water at 37 °C, determined by mercury porosimetry, *n* = 3. The measured porosity was 52%.

Originally, the paste was expected to set to struvite (MgNH_4_PO_4_·6H_2_O) and newberyite (MgHPO_4_·3H_2_O), similar to the “classical” struvite cement. However, after setting in water for 3 days, the paste consisted not only of farringtonite (Mg_3_(PO_4_)_2_), struvite, and newberyite, rather an additional formation of 12–39% dittmarite (MgNH_4_PO_4_·H_2_O) was observed (**Figure** **6**A). Due to the use of glycerol as a cement liquid and the slow exchange for water during setting, much less water is available in the system, which probably leads to the partial formation of the phase with a lower crystal water content, that is, dittmarite instead of struvite. According to Sarkar,^[^ ^49^ ^]^ hydration of dittmarite at room temperature leads to struvite formation. Indeed, the cement contained more struvite after 7 days compared to 3 days of setting in water, but dittmarite was also still present in the samples. The farringtonite content slightly increased with increasing content of fine TMP from 42% to 50%. At the same time, the struvite content strongly decreased with increasing content of fine TMP from 38% to 4%, whereas the dittmarite content strongly increased from 12% to 39%. This was surprising, because the finer TMP has a higher reactivity compared to the coarse TMP due to a higher surface‐volume ratio and a higher amorphous TMP content.^[^ ^50^ ^]^ It might be that the finer particles hampered the diffusion of water into the paste, which caused the lower conversion degree and the higher amount of dittmarite. Additionally, as already mentioned, the fine TMP might have reacted rapidly at the edge of the paste, which is in contact with water, creating a diffusion barrier.

Because the 20%‐paste exhibited a noticeably lower injection force compared to the other pastes, this one was selected as the optimum paste composition for further investigations. The pH value of the hardening 20%‐paste was rather alkaline, with values between 8.3 and 9.0 over a time course of 12 h (Figure S1, Supporting Information), possibly due to the excess of TMP and the presence of the finely ground TMP. Although the measured pH value is above the physiological condition (7.4), we did not observe any macroscopic signs of inflammation during the in vivo study (see Section 3.2, in vivo study). When the paste gets in contact with the humidity from the surrounding tissue, it likely hardens first at the contact area, that is, the rim of the paste. Thereby, the surrounding tissue is shielded from the core of the cement paste, and also protected from pH value changes within the cement paste core. Furthermore, the ongoing slow setting of the cement paste core likely allows balancing of pH value changes by the naturally occurring liquid exchange and buffering in the surrounding tissue. The pH value might also be shifted to more neutral conditions through partial or total replacement of the DAHP by ammonium dihydrogen phosphate.

**Figure 7:**
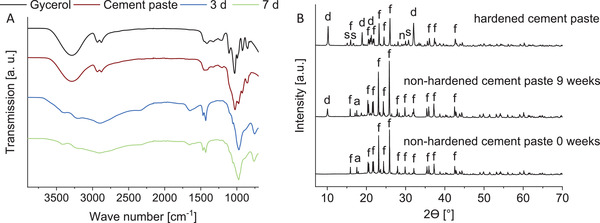
A) FTIR measurements of pure glycerol, the premixed 20%‐paste before setting, and the same paste after 3 or 7 days of setting in ultrapure water at 37 °C. The bands of the O—H bonds at 3300 cm^−1^ and of the C—H bonds at 2930 and 2880 cm^−1^ in glycerol were absent in the hardened cements. B) Phase composition determined by XRD of the premixed 20%‐paste without storage, after storage for 9 weeks, and after hardening for 3 days in ultrapure water at 37 °C (non‐stored paste). In addition to the farringtonite and DAHP peaks, dittmarite peaks were present in the paste stored for 9 weeks, indicating that the paste was already partly set. f‐farringtonite, d‐dittmarite, a‐DAHP, n‐newberyite.

The 52% porosity of the set 20%‐paste (Figure 6B) was noticeably higher compared to not only the 5–32% porosity^[^ ^12^ ^]^ of the “classical” cold‐setting struvite cement, but also higher compared to the prefabricated calcium magnesium phosphate paste from Ewald et al.,^[^ ^14^ ^]^ where the porosity was 2–26%. Assuming that the pores between the particles in the non‐hardened paste were completely filled with glycerol, the glycerol occupied 46% of the paste volume, calculated with the respective weight and theoretical density of each component (glycerol: 1.26 g cm^−3^, farringtonite: 2.8 g cm^−3^, DAHP: 1.62 g cm^−3^). In addition, 16% paste volume was filled with DAHP, which completely dissolved during the setting of the paste. According to Fourier transform infrared spectroscopy (FTIR) measurements, the bands of the O—H bonds at 3300 cm^−1^ and of the C—H bonds at 2930 and 2880 cm^−1^ in glycerol were absent in the hardened cements (**Figure** **7**A). This indicates that the glycerol was completely exchanged for water during setting. Furthermore, no DAHP peaks were present in the X‐ray diffraction (XRD) measurements after 3 days of setting in the water bath (Figure 7B). Together, the glycerol, which was exchanged for water over time, and the DAHP accounted for 62% by volume of the paste. However, dittmarite and struvite also have lower densities (2.2 and 1.7 g cm^−3^) compared to farringtonite (2.8 g cm^−3^), meaning that these phases occupy a higher volume for the same mass, which possibly reduces porosity. The higher density of dittmarite compared to struvite possibly also contributed to the higher porosity of the prefabricated cement paste compared to the “classical” struvite cement.

The majority of pores in the hardened cement exhibited a size between 10 nm and 10 µm (Figure 6B). In general, cold‐setting mineral cements, including calcium and magnesium phosphates, normally present an intrinsic microporosity due to their hardening mechanism.^[^ ^12^, ^51^ ^]^ The growth and entanglement of the crystals results in the formation of small pores, usually with a size below 1 µm.^[^ ^51^ ^]^ The presence of the large DAHP particles in the cement paste with a median diameter of 67 µm might have led to the observed small amount of large pores (>10 µm, Figure 6B), due to the complete dissolution of DAHP during hardening. Apparently, most of the pores created by the dissolution of the DAHP were filled with the newly formed mineral phases struvite, dittmarite, and newberyite. In vivo, larger pores (10–100 µm) are likely created in situ by the rapid dissolution of these high soluble phases, as already observed for struvite‐forming cements.^[^ ^17^ ^]^

**Figure 8:**
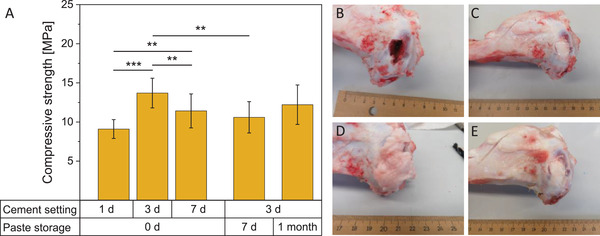
A) Compressive strength after different paste storage times and cement setting times (d‐days) of the paste with 20% fine TMP content, *n* ≥ 8. Compressive strength after setting was between 9 and 14 MPa. B) Pig tibia defect empty, C) filled with cement paste with 20% fine Mg_3_(PO_4_)_2_, D) covered with soft tissue, and E) 24 h after hardening at 37 °C. The cement hardened completely within 24 h in bone tissue. **p < 0.01 and ***p < 0.001.

The compressive strength of the 20%‐paste was 9 MPa after 1 day of setting in water and increased significantly to 14 MPa after 3 days (**Figure** **8**A). After 7 days of setting, the compressive strength was, with 11 MPa, slightly lower again. Therefore, the compressive strength was similar to the prefabricated Ca‐Mg‐paste with Miglyol (7–11 MPa),^[^ ^14^ ^]^ and considerably lower compared to the “classical” cold‐setting struvite cement, for which compressive strengths of 40–85 MPa were reported.^[^ ^12^, ^17^ ^]^ The compressive strength of the prefabricated cement was within the range of human cancellous bone (4–12 MPa).^[^ ^52^ ^]^ Due to the rather low compressive strength it is recommended for the use as void filler in non‐load‐bearing bone defects. The high porosity of 52% probably contributed to the observed low compressive strength. A lower glycerol content in the paste would probably reduce the porosity in the hardened cement, but also change the viscosity of the paste and likely increase injection force and impede injectability. An ex vivo test in pig tibia bone also demonstrated that the paste hardens not only in a water bath but also within 24 h in bone (Figure 8B–E).

Storage of the prefabricated paste for 1 or 4 weeks had only a minor influence on the compressive strength of the cement after setting (Figure 8A). The injection force increased significantly from 25 to 113 N when the paste was stored for 4 weeks (Figure 3A), presumably because minimal setting occurred due to a reaction with atmospheric moisture. Indeed, we observed the presence of dittmarite peaks in the XRD measurement in a 20%‐paste that was stored for 9 weeks (Figure 7B). Although the addition of fine TMP initially reduced the injection fore, it might be that it also reduced the storage stability because of its high reactivity. Due to the increase of viscosity and injection force during storage, the paste was not injected into the defect by syringe during the in vivo tests, rather directly implanted as a moldable mass by hand. Before implantation, the prefabricated pastes were stored 4–6 weeks, depending on the day of surgery. Additionally, we observed some minor phase separation of the dry components and glycerol during storage. Therefore, the paste was mixed after storage with a spatula before transferring to the syringe used for injection force/injectability measurement. For a possible translation into the clinics, the observed increase of viscosity and injection force during storage needs further optimization, for example, by including additional drying steps of the powder components and the glycerol before paste fabrication. For clinical use, a shelf life of at least 1 year would be desirable.

### 3.2 In Vivo Study

The surgeries proceeded without complications, except for one animal that had to be excluded from the study due to an anesthetic incident. The surgical application of the prefabricated cement paste was easy and the handling properties met the surgeon's needs. The animals recovered well post‐surgery and regained a physiological gait pattern within a few days. In the further course of the study, the sheep displayed no signs of discomfort or unphysiological movement and the surgical wounds displayed undisturbed wound healing.

#### 3.2.1 Macroscopical Observations

When the tibiae were collected for comprehensive post‐mortem analysis after 2 and 4 months, no macroscopical signs of tissue inflammation or infection were observed. The implanted cement pastes exhibited a good integration into the surrounding bone and a thin layer of newly formed bone covered the medial implant surface.

#### 3.2.2 Histological Evaluation

**Figure 9:**
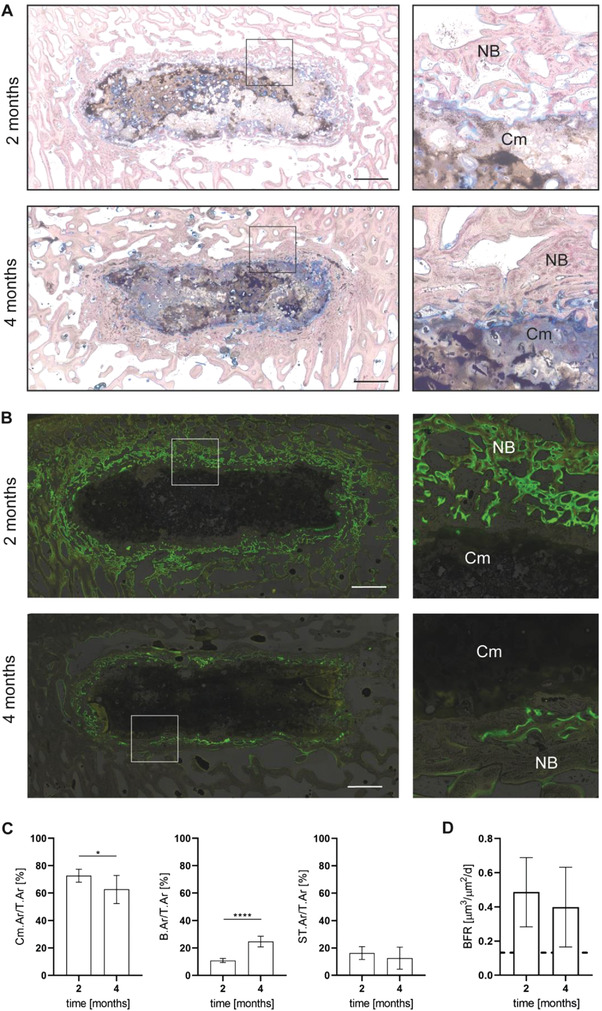
A) Representative histological images (Giemsa staining) and B) fluorescence images of the prefabricated cement paste (with 20% fine TMP content) at 2 or 4 months after implantation into mechanically loaded tibial bone defects in sheep. Overviews and magnifications of the bone‐implant interface. Scale bar: 2.5 mm. Cm: Cement paste, NB: newly formed bone. C) Histomorphometrical analyses: relative cement area per total tissue area (Cm.Ar/T.Ar), bone area per total tissue area (B.Ar/T.Ar), and soft tissue per total tissue area (ST.Ar/T.Ar), *p < 0.05, ****p < 0.0002, *n* = 6 for 2 months, *n* = 7 for 4 months. D) Bone formation rate (BFR) of fluorescence‐labeled bone adjacent to the cement paste at 2 and 4 months after implantation, *n* = 6 for 2 months, *n* = 7 for 4 months. Dashed line = BFR of surrounding intact trabecular bone.

Similarly on a histological level, no signs of inflammation or implant rejection were observed at either of the two implantation time points (**Figure** **9**A). This emphasizes the good biocompatibility of the implanted cement paste and is in accordance with the findings of other studies investigating magnesium phosphate‐based bone substitutes both in vitro and in bone defects in vivo.^[^ ^16^, ^17^, ^18^, ^20^, ^44^, ^53^, ^54^ ^]^ In all of these studies, a good biocompatibility of the magnesium phosphates was reported.

Qualitative histological analysis showed that the cement paste was continuously degraded during the implantation time, from the outer margins of the implant to the inside (Figure 9A). Simultaneously, the degrading cement paste was replaced by newly formed bone, which was lined by palisades of active osteoid‐producing osteoblasts (Figure S2, Supporting Information), resulting in a good and over time increasing osseointegration of the implanted cement paste. In addition to osteoblasts, osteoblastic precursor cells and newly formed blood vessels as well as phagocytes involved with the debridement of cement particles were observed at the resorption front. Released cement paste particles were embedded into the newly formed bone and were also found in the bone marrow between the emerging new bone trabeculae. Intensive osteoid production was observed close to the cement surface and over time, calcification of the osteoid was seen, indicating bone maturation. The degrading cement paste displayed a porous appearance and an inhomogeneous staining inside (Figure 9A), likely due to the ongoing degradation process. The margins of the implant appeared uneven and dispersed and new bone was formed in pores at the outer regions of the implant, evolving from the continuous resorption of the cement paste.

**Figure 10:**
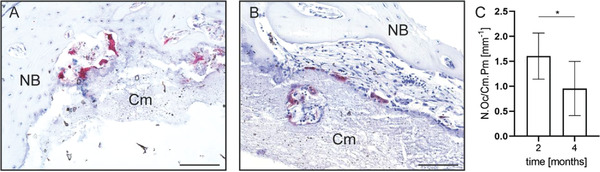
Representative histological images of paraffin sections stained for TRAP of the tibial bone defects treated with the cement paste at A) 2 months and B) 4 months after implantation. Cm: Cement paste. NB: newly formed bone, 200× magnification. Scale bar: 100 µm. C) Quantitative analysis of osteoclasts located on the cement surface, indicating active resorption. N.Oc/Cm.Pm: osteoclast number per cement perimeter. *p < 0.05, *n* = 6 for 2 months, *n* = 7 for 4 months.

Magnesium phosphate cements are resorbed by a combination of active cellular resorption and passive dissolution.^[^ ^15^ ^]^ In the present study, osteoclasts were observed on the surface of the cement paste at both investigation time points in paraffin sections stained for tartrate‐resistant acid phosphatase (TRAP), indicating active cellular implant resorption (**Figure** **10**A,B). The number of osteoclasts on the cement paste surface was significantly decreased between 2 and 4 months (Figure 10C), suggesting a decline in cell‐mediated degradation over time.

The findings of the qualitative histological analysis were supported by histomorphometrical analysis, revealing a significant decrease in the relative amount of residual cement over time, with a cement area per total tissue area (Cm.Ar/T.Ar) of 73% at 2 months and 63% at 4 months post‐surgery (Figure 9C). This was accompanied by a significant increase in the relative amount of newly formed bone, with a bone area per total tissue area (B.Ar/T.Ar) of 11% at 2 months and 25% at 4 months after implantation. Concomitantly, the relative amount of soft tissue (soft tissue area per total tissue area; ST.Ar/T.Ar) displayed a trend to decrease between 2 and 4 months from 16% to 13%, but without a significant difference between these two evaluation time points. Ideally, a bone cement is replaced by newly formed bone within a suitable time period without a temporary decline in biomechanical competence. A too slow or too rapid resorption impedes bone regeneration both biologically and biomechanically. Therefore, it is important to control the degradation behavior and adjust it to different clinical demands, which remains a major challenge in the development of degradable bone substitutes. The prefabricated cement paste investigated in the present study exhibited a rather rapid degradation rate compared to a calcium‐deficient hydroxyapatite (CDHA) control that was investigated in the same implantation model in an earlier study by Kanter et al.^[^ ^18^ ^]^ In this study, under similar experimental conditions as in the present study, the CDHA cement was well osseointegrated 10 months after implantation, but showed basically no degradation and no new bone formation.^[^ ^18^ ^]^

This underlines the accelerated degradation behavior of magnesium phosphate cements compared to calcium phosphate cements and might be attributed to a higher solubility of the struvite phase under physiological conditions. In the present study, the rapid degradation of the struvite cement paste did not exceed the new bone formation rate (BFR) and was well adjusted to bone regeneration, because no fibrous tissue formation occurred at the bone‐implant interface. In comparison to non‐prefabricated struvite cements in other studies that were implanted in the same defect model,^[^ ^18^, ^44^ ^]^ the prefabricated cement paste displayed a slightly increased or comparable degradation. In these studies, a struvite cement at a PLR of 1 or 2 g mL^−1^ displayed a relative cement area of 75%^[^ ^44^ ^]^ and 64%^[^ ^18^ ^]^ at 4 months after implantation, respectively. The prefabricated paste exhibited a relative cement area of 63% at 4 months post‐surgery. The farringtonite content was with 46–53% (depending on the paste storage time before setting) between the non‐prefabricated struvite cements, which was 37% for a PLR of 1 g mL^−1^ and 60% for a PLR of 2 g mL^−1^. According to fertilizer research, struvite and dittmarite have a similar solubility in water or a tris(hydroxymethyl)aminomethane‐hydrochloric acid buffer (pH 7).^[^ ^55^, ^56^ ^]^ Additionally, transformation of dittmarite to struvite during hydration at room temperature was reported, which would contribute to a similar solubility.^[^ ^49^ ^]^ However, XRD measurements of implant remnants after 2 months indicated that the dittmarite phase was not completely transformed to struvite, although the dittmarite phase could be reliably detected only in one of the three investigated 2‐month samples (see Section 3.2.3, phase composition of explants). The higher porosity of 52% compared to 7–12%^[^ ^18^, ^44^ ^]^ of the non‐prefabricated struvite cements did not lead to a great increase of the degradation speed, possibly because the pore size was also in the lower micrometer range (Figure 6B). The degradation of the prefabricated cement paste was, moreover, accompanied by a higher relative amount of newly formed bone 4 months post‐surgery (25%) compared to the non‐prefabricated struvite cements, showing a relative bone area of 13%^[^ ^44^ ^]^ at a PLR of 1 g mL^−1^ and 17%^[^ ^18^ ^]^ at a PLR of 2 g mL^−1^. By contrast, for the CDHA reference cement from the previous study no new bone formation was observed after 10 months, due to the very limited degradation.^[^ ^18^ ^]^ This is in accordance with other in vivo studies in large animal models reporting a slow degradation and only limited new bone formation of HA‐forming cements.^[^ ^57^, ^58^, ^59^, ^60^, ^61^, ^62^, ^63^, ^64^ ^]^

For brushite cements generally a faster degradation compared to HA was observed in vivo in large animal models. Flautre et al. reported 64% remaining cement and 28% new bone formation 3 months after implantation of a brushite cement in drill hole femur defects in sheep, which is similar to the results of the prefabricated cement in the present study.^[^ ^65^ ^]^ Theiss et al.^[^ ^66^ ^]^ and Apelt et al.^[^ ^57^ ^]^ observed a very rapid degradation of the brushite cement chronOS Inject (Synthes, US) in drill hole femur/humerus defects in sheep, with 38% residual cement 2 months after implantation^[^ ^66^ ^]^ and 22% residual cement 4 months after implantation.^[^ ^57^ ^]^ However, the initially very rapid degradation observed in the study of Apelt et al.^[^ ^57^ ^]^ slowed down after 4 months: 19% remaining cement was present 6 months after implantation, due to the conversion of brushite into HA and the stability of the ß‐TCP granules.^[^ ^24^, ^57^ ^]^ Apparently the addition of 5 wt% magnesium hydrogen phosphate trihydrate in the cement composition retarded the conversion to the low soluble calcium phosphate, because magnesium ions act as an inhibitor for apatite formation, but could not completely prevent it.^[^ ^24^, ^57^ ^]^ Grover et al.^[^ ^67^ ^]^ successfully improved the in vivo degradation speed of a brushite cement by modification with 28 wt% amorphous calcium pyrophosphate in the set cement, which likely also prevented the formation of low crystalline HA. But still both cements, the reference brushite cement and the pyrophosphate modified brushite cement, exhibited a rather slow degradation in drill hole tibia defects in sheep. After 3 months, only 5–6% new bone formation was observed, and after 12 months 14–33% new bone was found with large cement parts still being present at the implantation site.^[^ ^67^ ^]^ By contrast, for the non‐prefabricated struvite cement from our previous study,^[^ ^18^ ^]^ implanted in the same animal and defect model as in the present study, a residual cement of ≈3% was observed 10 months after implantation and the defect was filled with new trabecular bone.^[^ ^18^ ^]^ Because of the shorter implantation time points and the slightly different phase composition of the cement due to the presence of dittmarite in the current study, no statement can be done regarding the long‐term degradation of the prefabricated paste. However, based on the similarity between struvite and dittmarite and the comparable rates of the degradation 4 months after implantation between the prefabricated and non‐prefabricated struvite cements, we assume that the long‐term degradation is similar.

Gefel et al.^[^ ^68^ ^]^ recently observed the formation of an amorphous or low crystalline HA layer on 3D printed struvite scaffolds after immersion in simulated body fluid, which is a necessary, but not a sufficient condition for osteoinductivity.^[^ ^69^ ^]^ Furthermore, in the recently published study by Kaiser et al.,^[^ ^44^ ^]^ we were able to observe heterotopic bone formation after 4 months in prehardened struvite cement samples that were implanted subcutaneously (s.c.), indicating osteoinductivity. It is likely that the prefabricated cement paste in the current study has a similar osteoinductive potential.

Qualitative fluorescence microscopy showed that primarily yellow tetracycline‐labeled bone was present directly on the cement surface, followed by green calcein‐labeled bone, suggesting that newly formed bone was deposited on earlier formed bone and not on the remaining uncovered implant (Figure 9B). Overall, the newly formed bone replacing the degrading cement displayed an increased intensity of the yellow tetracycline and green calcein fluorescence compared to the surrounding trabecular tibial bone. These observations were supported by an increased BFR of the newly formed bone adjacent to the cement surface compared to that of the intact tibial bone (Figure 9D). Between 2 and 4 months, the BFR showed a trend to decrease, however, without reaching significant difference in contrast to a recent study of our group investigating a struvite cement.^[^ ^44^ ^]^ The trend over time of a decreasing BFR of the newly formed bone taken together with the significant decrease in the number of osteoclasts located on the cement surface suggested that the overall degradation and new BFR decreases with an increasing osseointegration of the implant.

Although a quantitative biomechanical evaluation was not performed, the cement paste exhibited a good performance in the used moderately loaded defect model. No fissures or crack formation of the cement paste were observed after implantation, while bone regeneration was enabled in a simultaneous degradation and new bone formation process. This suggests that the cement paste provided sufficient mechanical stability in the current defect model, despite the fact that the in vitro compressive strength of the hardened prefabricated paste of 9–14 MPa was considerably lower compared to the classic non‐prefabricated struvite cements, which exhibit compressive strengths of 40–85 MPa.^[^ ^12^, ^17^ ^]^

#### 3.2.3 Phase Composition of Explants

**Figure 11:**
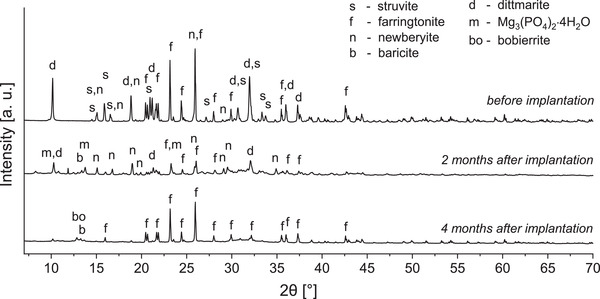
Phase composition determined by XRD before and 2 and 4 months after implantation. The samples “before implantation” were prepared by setting of the 20%‐paste for 3 days in water at 37 °C. After 4 months, mainly farringtonite and TMP hydrates remained.

XRD measurements of cement remnants revealed that after 2 months the material consisted mainly of newberyite, farringtonite, Mg_3_(PO_4_)_2_·4H_2_O, and dittmarite (**Figure** **11**). The dittmarite and struvite phases appeared to be degraded rapidly, because very limited amounts of dittmarite and struvite were found after 2 months, and no dittmarite or struvite could be detected after 4 months. After 2 months and in one sample after 4 months (Figure S3, Supporting Information), high amounts of a hydrated magnesium phosphate phase (Mg_3_(PO_4_)_2_·4H_2_O) were found, possibly due to the reaction of farringtonite with humidity coming from the surrounding tissue. After 4 months, the results were different between the measured samples. In one case, mainly farringtonite with small amounts of bobierrite and baricite (Mg_3_(PO_4_)_2_·8H_2_O) remained (Figure 11). In another sample, the remaining material consisted mainly of Mg_3_(PO_4_)_2_·4H_2_O, newberyite, and farringtonite, similar to the composition after 2 months (Figure S3, Supporting Information). It might be that a slightly different presence of water/humidity leads to a varying degree of hydration of the farringtonite, resulting in the observed differences in the phase composition. Biologically, these differences should not have any influence, because all phases (farringtonite, its hydrates, and newberyite) are biocompatible.^[^ ^70^, ^71^, ^72^ ^]^ Additionally, the solubility of farringtonite and bobierrite, for example, is very similar.^[^ ^13^ ^]^ In the third sample, mainly HA was found, but here very little of the material remained, such that bone was possibly also included in the prepared explant sample (Figure S3, Supporting Information).

## 4 Conclusion

Building on the reported rapid degradation and bone regeneration of struvite‐forming cements, we developed an injectable, prefabricated magnesium ammonium phosphate bone cement that can be directly applied during surgery. The cement paste was based on glycerol, which is exchanged against water coming from the surrounding tissue, resulting in a rapid hardening of the cement within 24 h. A trimodal PSD of the raw powders in the cement paste proved to be advantageous regarding the injection force, due to a higher packing density of the raw powder and thereby increased flowability. The application as a prefabricated paste did not alter the good biocompatibility reported for struvite bone cements. No macroscopical or histological signs of inflammation were detected after implantation in partly loaded tibia defects in an ovine model. In contrast to an established HA cement, which displayed basically no degradation even at 10 months after implantation in the same implantation model,^[^ ^18^ ^]^ 37% of the cement were degraded after 4 months, and 25% of the original implant were replaced by new bone tissue. We conclude that the prefabricated paste may be suitable for the treatment of trabecular bone defects in non‐loaded clinical situations, promotes new bone formation in a high quantity, and greatly improves the application during surgery. For a potential clinical use, further optimization regarding the storage stability of the prefabricated paste is necessary.

## Conflict of Interest

The authors declare no conflict of interest.

## Supporting information

Supporting Information

## Data Availability

The data that support the findings of this study are available from the corresponding author upon reasonable request.

## References

[1] B. Wildemann , A. Ignatius , F. Leung , L. A. Taitsman , R. M. Smith , R. Pesantez , M. J. Stoddart , R. G. Richards , J. B. Jupiter , Nat. Rev. Dis. Primers 2021, 7, 57. 34354083 10.1038/s41572-021-00289-8

[2] R. Dimitriou , E. Jones , D. McGonagle , P. V. Giannoudis , BMC Med. 2011, 9, 66. 21627784 10.1186/1741-7015-9-66 PMC3123714

[3] A. H. Schmidt , Injury 2021, 52, S18. 10.1016/j.injury.2021.01.043 33563416

[4] M. Bohner , B. L. G. Santoni , N. Dobelin , Acta Biomater. 2020, 113, 23. 32565369 10.1016/j.actbio.2020.06.022

[5] A. M. Yousefi , J. Appl. Biomater. Funct. Mater. 2019, 17, 2280800019872594. 31718388 10.1177/2280800019872594

[6] L. Schroter , F. Kaiser , S. Stein , U. Gbureck , A. Ignatius , Acta Biomater. 2020, 117, 1. 32979583 10.1016/j.actbio.2020.09.031

[7] R. Klein , R. Tetzlaff , C. Weiss , M. K. Schafer , M. Tanner , B. Wiedenhofer , I. Grafe , P. J. Meeder , G. Noeldge , P. P. Nawroth , C. Kasperk , Clin. Spine Surg. 2017, 30, E291. 28323714 10.1097/BSD.0b013e3182aab2df

[8] W. Linhart , D. Briem , M. Amling , J. M. Rueger , J. Windolf , Unfallchirurg 2004, 107, 154. 14999381 10.1007/s00113-003-0707-5

[9] E. Roddy , M. R. DeBaun , A. Daoud‐Gray , Y. P. Yang , M. J. Gardner , Eur. J. Orthop. Surg. Traumatol. 2018, 28, 351. 29080923 10.1007/s00590-017-2063-0

[10] G. Chandra , A. Pandey , Probl. Biocybern. Biomed. Eng. 2020, 40, 596.

[11] H. Zhou , B. Liang , H. Jiang , Z. Deng , K. Yu , J. Magnesium Alloys 2021, 9, 779.

[12] N. Ostrowski , A. Roy , P. N. Kumta , ACS Biomater. Sci. Eng. 2016, 2, 1067. 33445235 10.1021/acsbiomaterials.6b00056

[13] M. Nabiyouni , T. Bruckner , H. Zhou , U. Gbureck , S. B. Bhaduri , Acta Biomater. 2018, 66, 23. 29197578 10.1016/j.actbio.2017.11.033

[14] A. Ewald , D. Kreczy , T. Bruckner , U. Gbureck , M. Bengel , A. Hoess , B. Nies , J. Bator , U. Klammert , A. Fuchs , Materials 2019, 12, 2119. 31266228 10.3390/ma12132119 PMC6651064

[15] C. Grossardt , A. Ewald , L. M. Grover , J. E. Barralet , U. Gbureck , Tissue Eng., Part A 2010, 16, 3687. 20673025 10.1089/ten.TEA.2010.0281

[16] F. Wu , J. Wei , H. Guo , F. Chen , H. Hong , C. Liu , Acta Biomater. 2008, 4, 1873. 18662897 10.1016/j.actbio.2008.06.020

[17] B. Kanter , M. Geffers , A. Ignatius , U. Gbureck , Acta Biomater. 2014, 10, 3279. 24769112 10.1016/j.actbio.2014.04.020

[18] B. Kanter , A. Vikman , T. Bruckner , M. Schamel , U. Gbureck , A. Ignatius , Acta Biomater. 2018, 69, 352. 29409867 10.1016/j.actbio.2018.01.035

[19] J. A. Kim , J. Lim , R. Naren , H. S. Yun , E. K. Park , Acta Biomater. 2016, 44, 155. 27554019 10.1016/j.actbio.2016.08.039

[20] Y. Yu , J. Wang , C. Liu , B. Zhang , H. Chen , H. Guo , G. Zhong , W. Qu , S. Jiang , H. Huang , Colloids Surf., B 2010, 76, 496. 10.1016/j.colsurfb.2009.12.010 20074920

[21] F. Tamimi , Z. Sheikh , J. Barralet , Acta Biomater. 2012, 8, 474. 21856456 10.1016/j.actbio.2011.08.005

[22] Z. Sheikh , Y. L. Zhang , L. Grover , G. E. Merle , F. Tamimi , J. Barralet , Acta Biomater. 2015, 26, 338. 26300333 10.1016/j.actbio.2015.08.031

[23] G. Penel , N. Leroy , P. Van Landuyt , B. Flautre , P. Hardouin , J. Lemaitre , G. Leroy , Bone 1999, 25, 81S. 10458282 10.1016/s8756-3282(99)00139-8

[24] M. Bohner , F. Theiss , D. Apelt , W. Hirsiger , R. Houriet , G. Rizzoli , E. Gnos , C. Frei , J. A. Auer , B. von Rechenberg , Biomaterials 2003, 24, 3463. 12809775 10.1016/s0142-9612(03)00234-5

[25] M. P. Ginebra , M. Espanol , E. B. Montufar , R. A. Perez , G. Mestres , Acta Biomater. 2010, 6, 2863. 20123046 10.1016/j.actbio.2010.01.036

[26] H. H. Xu , L. E. Carey , C. G. Simon Jr. , S. Takagi , L. C. Chow , Dent. Mater. 2007, 23, 433. 16678895 10.1016/j.dental.2006.02.014 PMC2646467

[27] L. E. Carey , H. H. Xu , C. G. Simon Jr. , S. Takagi , L. C. Chow , Biomaterials 2005, 26, 5002. 15769536 10.1016/j.biomaterials.2005.01.015 PMC2645070

[28] L. C. Chow , S. Takagi , (ADA Foundation, Chicago, IL), US 9,259,439 B2, 2006.

[29] A. Sugawara , L. C. Chow , S. Takagi , H. Chohayeb , J. Endod. 1990, 16, 162. 2074405 10.1016/S0099-2399(06)81963-1

[30] S. Takagi , L. C. Chow , S. Hirayama , A. Sugawara , J. Biomed. Mater. Res., Part B 2003, 67, 689. 10.1002/jbm.b.10065 14598395

[31] S. Heinemann , S. Rossler , M. Lemm , M. Ruhnow , B. Nies , Acta Biomater. 2013, 9, 6199. 23261920 10.1016/j.actbio.2012.12.017

[32] A. Lode , C. Heiss , G. Knapp , J. Thomas , B. Nies , M. Gelinsky , M. Schumacher , Acta Biomater. 2018, 65, 475. 29107056 10.1016/j.actbio.2017.10.036

[33] J. Weichhold , F. Goetz‐Neunhoeffer , K. Hurle , U. Gbureck , Ceram. Int. 2022, 48, 15390.

[34] B. Han , P. W. Ma , L. L. Zhang , Y. J. Yin , K. D. Yao , F. J. Zhang , Y. D. Zhang , X. L. Li , W. Nie , Acta Biomater. 2009, 5, 3165. 19427931 10.1016/j.actbio.2009.04.024

[35] J. Luo , H. Engqvist , C. Persson , Acta Biomater. 2018, 81, 304. 30291976 10.1016/j.actbio.2018.10.001

[36] J. Engstrand , J. Aberg , H. Engqvist , Mater. Sci. Eng., C 2013, 33, 527. 10.1016/j.msec.2012.09.026 25428105

[37] J. Engstrand , C. Persson , H. Engqvist , Biomatter 2013, 3, e1. 10.4161/biom.27249 PMC3891756 24270588

[38] A. Ignatius , K. Unterricker , K. Wenger , M. Richter , L. Claes , P. Lohse , H. Hirst , J. Mater. Sci.: Mater. Med. 1997, 8, 753. 15348785 10.1023/a:1018508511787

[39] U. Simon , P. Augat , A. Ignatius , L. Claes , J. Biomech. 2003, 36, 1079. 12831732 10.1016/s0021-9290(03)00114-3

[40] S. Reitmaier , A. Kovtun , J. Schuelke , B. Kanter , M. Lemm , A. Hoess , S. Heinemann , B. Nies , A. Ignatius , J. Orthop. Res. 2018, 36, 106. 28574614 10.1002/jor.23623

[41] A. Ignatius , M. Peraus , S. Schorlemmer , P. Augat , W. Burger , S. Leyen , L. Claes , Biomaterials 2005, 26, 2325. 15585235 10.1016/j.biomaterials.2004.07.029

[42] K. Donath , G. Breuner , J. Oral Pathol. 1982, 11, 318. 6809919 10.1111/j.1600-0714.1982.tb00172.x

[43] D. W. Dempster , J. E. Compston , M. K. Drezner , F. H. Glorieux , J. A. Kanis , H. Malluche , P. J. Meunier , S. M. Ott , R. R. Recker , A. M. Parfitt , J. Bone Miner. Res. 2013, 28, 2.23197339

[44] F. Kaiser , L. Schroter , S. Stein , B. Kruger , J. Weichhold , P. Stahlhut , A. Ignatius , U. Gbureck , Acta Biomater. 2022, 145, 358. 35443213 10.1016/j.actbio.2022.04.019

[45] R. O'Neill , H. O. McCarthy , E. B. Montufar , M. P. Ginebra , D. I. Wilson , A. Lennon , N. Dunne , Acta Biomater. 2017, 50, 1. 27838464 10.1016/j.actbio.2016.11.019

[46] M. Bohner , G. Baroud , Biomaterials 2005, 26, 1553. 15522757 10.1016/j.biomaterials.2004.05.010

[47] M. Habib , G. Baroud , F. Gitzhofer , M. Bohner , Acta Biomater. 2008, 4, 1465. 18445539 10.1016/j.actbio.2008.03.004

[48] G. Baroud , E. Cayer , M. Bohner , Acta Biomater. 2005, 1, 357. 16701814 10.1016/j.actbio.2005.01.003

[49] A. K. Sarkar , J. Mater. Sci. 1991, 26, 2514.

[50] T. Brückner , K. Hurle , A. Stengele , J. Groll , U. Gbureck , J. Am. Ceram. Soc. 2018, 101, 1830.

[51] I. Lodoso‐Torrecilla , J. van den Beucken , J. A. Jansen , Acta Biomater. 2021, 119, 1. 33065287 10.1016/j.actbio.2020.10.013

[52] A. J. W. Johnson , B. A. Herschler , Acta Biomater. 2011, 7, 16. 20655397 10.1016/j.actbio.2010.07.012

[53] S. A. Schendel , J. Peauroi , J. Craniofacial Surg. 2009, 20, 461. 10.1097/SCS.0b013e31819b9819 19305245

[54] A. Ewald , K. Helmschrott , G. Knebl , N. Mehrban , L. M. Grover , U. Gbureck , J. Biomed. Mater. Res., Part B 2011, 96, 326. 10.1002/jbm.b.31771 21210513

[55] G. L. Bridger , M. L. Salutsky , R. W. Starostka , J. Agric. Food Chem. 1962, 10, 181.

[56] M. S. Massey , J. G. Davis , J. A. Ippolito , R. E. Sheffield , Agron. J. 2009, 101, 323.

[57] D. Apelt , F. Theiss , A. O. El‐Warrak , K. Zlinszky , R. Bettschart‐Wolfisberger , M. Bohner , S. Matter , J. A. Auer , B. von Rechenberg , Biomaterials 2004, 25, 1439. 14643619 10.1016/j.biomaterials.2003.08.073

[58] H. Kobayashi , T. Fujishiro , S. M. Belkoff , N. Kobayashi , A. S. Turner , H. B. Seim 3rd , J. Zitelli , M. Hawkins , T. W. Bauer , J. Biomed. Mater. Res., Part A 2009, 88, 880. 10.1002/jbm.a.31933 18381636

[59] R. D. Welch , B. H. Berry , K. Crawford , H. Zhang , M. Zobitz , D. Bronson , S. Krishnan , J. Orthop. Res. 2002, 20, 464. 12038619 10.1016/S0736-0266(01)00124-3

[60] T. M. Turner , R. M. Urban , K. Singh , D. J. Hall , S. M. Renner , T. H. Lim , M. J. Tomlinson , H. S. An , Spine J. 2008, 8, 482. 18455113 10.1016/j.spinee.2006.12.007

[61] C. K. G. Spies , S. Schnürer , T. Gotterbarm , S. J. Breusch , J. Appl. Biomater. Biomech. 2010, 8, 175. 21337309

[62] N. W. Kent , G. Blunn , N. Karpukhina , G. Davis , R. F. de Godoy , R. M. Wilson , M. Coathup , L. Onwordi , W. Y. Quak , R. Hill , J. Biomed. Mater. Res., Part B 2018, 106, 21. 10.1002/jbm.b.33809 29218858

[63] E. Verron , M. L. Pissonnier , J. Lesoeur , V. Schnitzler , B. H. Fellah , H. Pascal‐Moussellard , P. Pilet , O. Gauthier , J. M. Bouler , Acta Biomater. 2014, 10, 4887. 25050773 10.1016/j.actbio.2014.07.012

[64] L. A. Galovich , A. Perez‐Higueras , J. R. Altonaga , J. M. Orden , M. L. Barba , M. T. Morillo , Eur. Spine J. 2011, 20, 376. 21773815 10.1007/s00586-011-1905-4 PMC3175823

[65] B. Flautre , C. Delecourt , M. C. Blary , P. Van Landuyt , J. Lemaitre , P. Hardouin , Bone 1999, 25, 35S. 10458272 10.1016/s8756-3282(99)00147-7

[66] F. Theiss , D. Apelt , B. Brand , A. Kutter , K. Zlinszky , M. Bohner , S. Matter , C. Frei , J. A. Auer , B. von Rechenberg , Biomaterials 2005, 26, 4383. 15701367 10.1016/j.biomaterials.2004.11.056

[67] L. M. Grover , A. J. Wright , U. Gbureck , A. Bolarinwa , J. Song , Y. Liu , D. F. Farrar , G. Howling , J. Rose , J. E. Barralet , Biomaterials 2013, 34, 6631. 23747007 10.1016/j.biomaterials.2013.05.001

[68] E. Gefel , C. Moseke , A.‐M. Schmidt , N. Dümmler , P. Stahlhut , A. Ewald , A. Meyer‐Lindenberg , E. Vorndran , Bioact. Mater. 2023, 19, 376. 35633870 10.1016/j.bioactmat.2022.05.014 PMC9127104

[69] M. Bohner , R. J. Miron , Mater. Today 2019, 22, 132.

[70] N. Golafshan , E. Vorndran , S. Zaharievski , H. Brommer , F. B. Kadumudi , A. Dolatshahi‐Pirouz , U. Gbureck , R. van Weeren , M. Castilho , J. Malda , Biomaterials 2020, 261, 120302. 32932172 10.1016/j.biomaterials.2020.120302 PMC7116184

[71] F. C. M. Driessens , M. G. Boltong , M. I. Zapatero , R. M. H. Verbeeck , W. Bonfield , O. Brmdez , E. Fernndez , M. P. Ginebra , J. A. Planell , J. Mater. Sci.: Mater. Med. 1995, 6, 272.

[72] U. Klammert , A. Ignatius , U. Wolfram , T. Reuther , U. Gbureck , Acta Biomater. 2011, 7, 3469. 21658480 10.1016/j.actbio.2011.05.022

