# Supplementary material for: Ready‐To‐Use and Rapidly Biodegradable Magnesium Phosphate Bone Cement: In Vivo Evaluation in Sheep
Source: Adv Healthc Mater. 2023 Jun 4;12(26):2300914. doi: 10.1002/adhm.202300914 (PMC11468836; doi:10.1002/adhm.202300914)
Supplement: Supplementary file 1 — Supporting Information [file ADHM-12-2300914-s001.pdf]

ADVANCED HEALTHCARE MATERIALS

Supporting Information

for *Adv. Healthcare Mater.*, DOI 10.1002/adhm.202300914

Ready-To-Use and Rapidly Biodegradable Magnesium Phosphate Bone Cement: In Vivo Evaluation in Sheep

Lena Schröter, Friederike Kaiser, Anna-Lena Preißler, Philipp Wohlfahrt, Oliver Küppers, Uwe Gbureck and Anita Ignatius**

Supporting Information

Ready-to-Use and Rapidly Biodegradable Magnesium Phosphate Bone Cement: In Vivo Evaluation in Sheep

Lena Schröter[#], Friederike Kaiser[#], Anna-Lena Preißler, Philipp Wohlfahrt, Oliver Küppers, Uwe Gbureck[$]*, Anita Ignatius[$]*

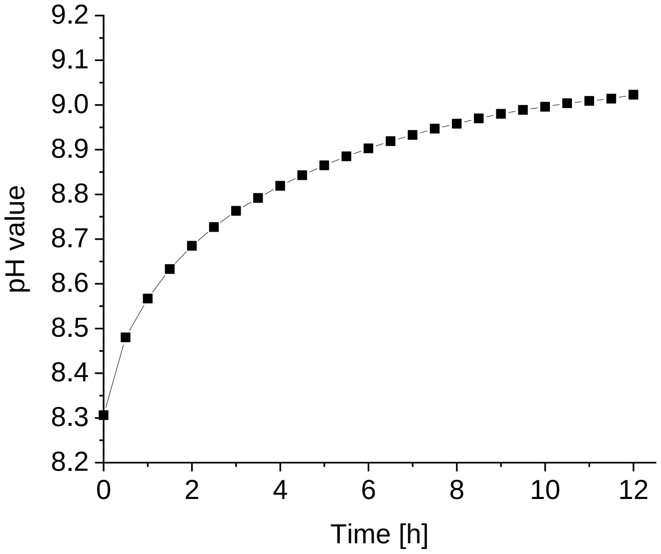

Figure S1. pH-value of the prefabricated paste with 20% fine TMP content measured for 12 hours within the hardening paste, overlayed by phosphate buffered saline.

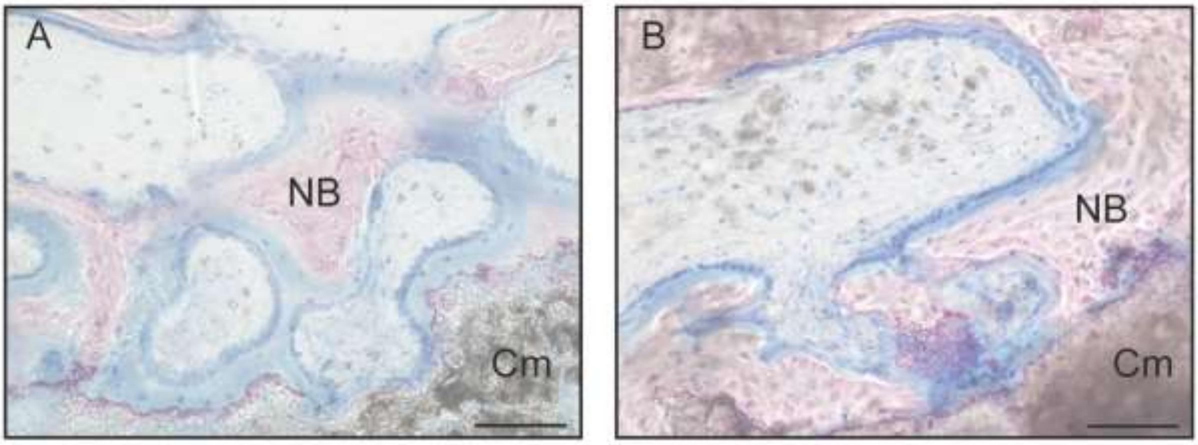

Figure S2. Representative histological images of the bone-implant interface of tibial defects treated with the prefabricated cement paste at A) 2 months and B) 4 months after implantation. New bone (NB) is deposited directly on the cement (Cm) surface. A) Intensive osteoid production by palisades of active osteoblasts 2 months after implantation. B) Increasing osseointegration of the prefabricated cement paste 4 months after implantation. Giemsa staining. 200 × magnification. Scale bar 100 μm.

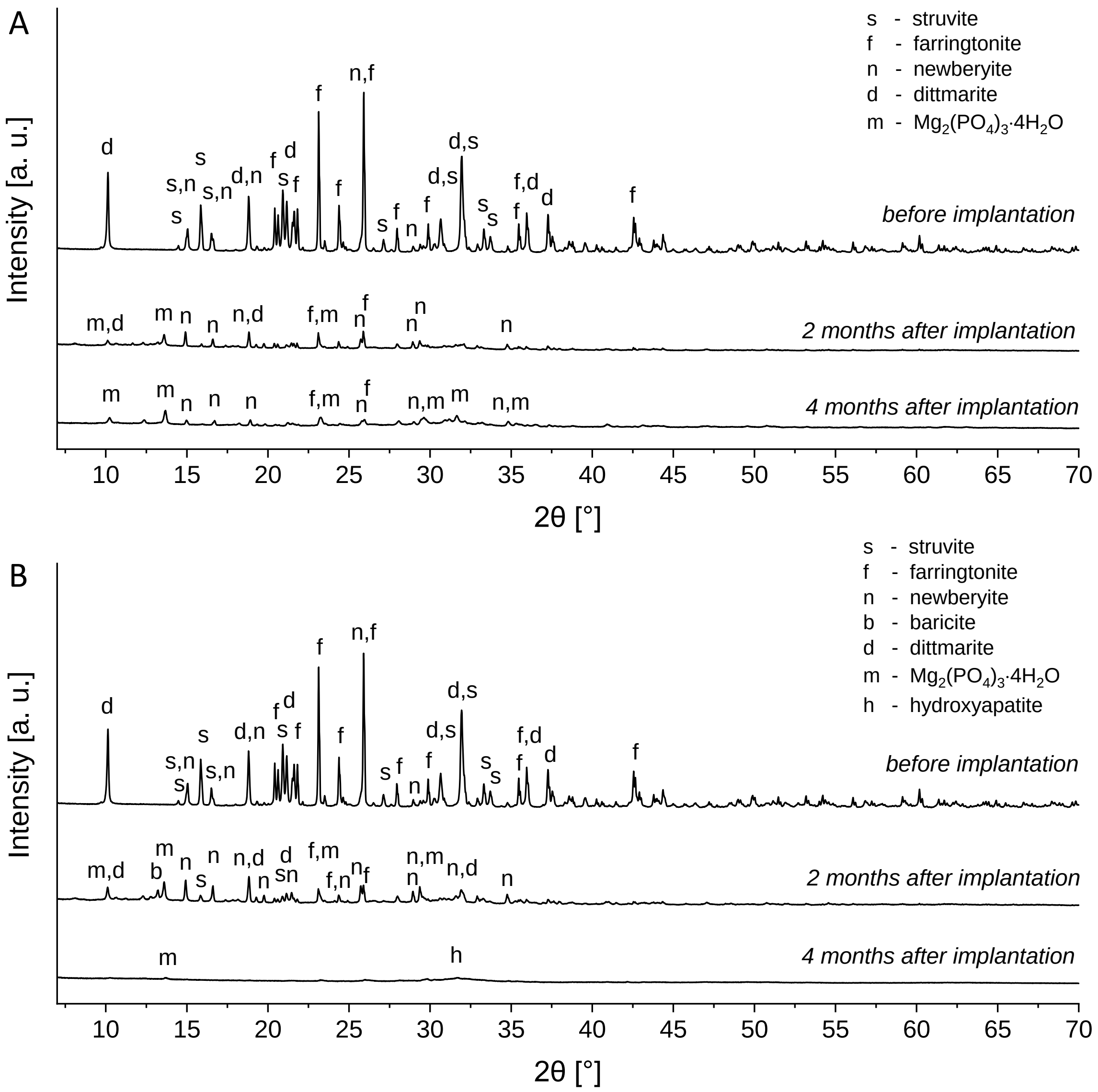

Figure S3. Phase composition determined by XRD before and 2 and 4 months after implantation. A and B contain measurements of different samples.
